# Using Oxidative Electrodes to Enrich Novel Members in the *Desulfobulbaceae* Family from Intertidal Sediments

**DOI:** 10.3390/microorganisms9112329

**Published:** 2021-11-11

**Authors:** Cheng Li, Clare E. Reimers, Yvan Alleau

**Affiliations:** College of Earth, Ocean, and Atmospheric Sciences, Oregon State University, Corvallis, OR 97331, USA; clare.reimers@oregonstate.edu (C.E.R.); yvan.alleau@oregonstate.edu (Y.A.)

**Keywords:** *Desulfobulbaceae*, bioelectrochemical reactor, electrogen, electrogenic sulfur oxidation, cable bacteria

## Abstract

Members in the family of *Desulfobulbaceae* may be influential in various anaerobic microbial communities, including those in anoxic aquatic sediments and water columns, and within wastewater treatment facilities and bioelectrochemical systems (BESs) such as microbial fuel cells (MFCs). However, the diversity and roles of the *Desulfobulbaceae* in these communities have received little attention, and large portions of this family remain uncultured. Here we expand on findings from an earlier study (Li, Reimers, and Alleau, 2020) to more fully characterize *Desulfobulbaceae* that became prevalent in biofilms on oxidative electrodes of bioelectrochemical reactors. After incubations, DNA extraction, microbial community analyses, and microscopic examination, we found that a group of uncultured *Desulfobulbaceae* were greatly enriched on electrode surfaces. These *Desulfobulbaceae* appeared to form filaments with morphological features ascribed to cable bacteria, but the majority were taxonomically distinct from recognized cable bacteria genera. Thus, the present study provides new information about a group of *Desulfobulbaceae* that can exhibit filamentous morphologies and respire on the oxidative electrodes. While the phylogeny of cable bacteria is still being defined and updated, further enriching these members can contribute to the overall understanding of cable bacteria and may also lead to identification of successful isolation strategies.

## 1. Introduction

Various microorganisms conserve energy by passing electrons over distances greater than their cell-length [1,2]. By tightly coupling electron donors and acceptors, mechanisms of direct extracellular electron transfer (DEET) grant microbial cells competitive advantages in locations where the diffusive transfer of chemical shuttles is limited. Currently, DEET mechanisms have been extensively characterized in several model microorganisms and systems, including *Geobacter*, a genus classified under the *Deltaproteobacteria*, *Shewanella*, a genus of *Gammaproteobacteria*, and the consortia of anaerobic methane-oxidizing archaea and sulfate-reducing bacteria [3,4,5]. Meanwhile, culture-independent molecular techniques such as catalyzed reporter deposition fluorescence in situ hybridization (CARD-FISH), 16S rRNA gene surveys, and metagenome analyses have suggested that there are many diverse microorganisms in a variety of environments that have the functional ability to perform DEET.

Among the list of DEET candidates are numerous members in the family of *Desulfobulbaceae*. Most described members of this family are recognized as sulfate reducing chemotrophs that can also divert electrons from assorted reduced compounds to a wide spectrum of electron acceptors other than oxygen [6,7,8]. The ability to utilize a versatile collection of electron acceptors to conserve energy has made members in the *Desulfobulbaceae* family important players in the biogeochemical cycles of various anoxic environments [9,10,11]. They thrive in natural anaerobic aquatic sediments and water bodies and in wastewater collected for biological treatment. Since the invention of bioelectrochemical systems (BESs) such as microbial fuel cells (MFCs), they can often be observed in anodic communities and biofilms [11,12,13]. However, the roles of the *Desulfobulbaceae* in these anoxic environments are not entirely clear, and a large portion of this family remains uncultured.

For example, a multi-genus group of long, multicellular, and filamentous *Desulfobulbaceae* known as “cable bacteria (CB)” has been recently discovered in the uppermost centimeters of marine, freshwater, and groundwater sediments [14,15,16,17,18,19]. By conducting a unique type of DEET through a parallel network of periplasmic fibers, CB catalyze spatially separated oxygen reduction (O_2_ + 4e^−^ + 4H^+^ → 2H_2_O) and sulfide oxidation (½ H_2_S + 2H_2_O → ½ SO_4_^2−^ + 4e^−^ + 5H^+^), a process referred to as electrogenic sulfur oxidation (e-SOx) [14,15]. The natural co-occurrence of e-SOx and CB has been documented in surficial sediments from assorted habitats, including intertidal mudflats, mangroves, rivers, salt marshes, groundwater aquifers, and certain bioturbated sediments exhibiting stable burrows produced by mud shrimp or parchment worms [14,20,21,22,23,24,25,26]. Since discovery, CB have gained considerable attention. Firstly, the two separated half-reactions impact the localized porewater pH, creating a pH maximum and a pH minimum, respectively, in oxic and sulfidic layers [16,19]. The two pH extremes then promote a series of secondary reactions that exert strong geochemical impacts on the elemental cycling of iron, manganese, calcium, phosphorus, and nitrogen [27,28,29,30,31,32]. Secondly, the periplasmic fibers of CB are highly conductive (up to 79 S/cm) which promises to inspire research into new bio-engineering applications [33,34]. Despite the attention that CB have gained, they remain uncultured outside of sediment.

A simple explanation behind the unculturability of various *Desulfobulbaceae* members, and perhaps of many other uncultured bacteria, is the unsuccessful laboratory replication of combinations of essential environmental conditions, including pH, temperature, nutrients, osmotic pressure, redox gradients, and solid substrates [35]. Another explanation is that the energy metabolism of certain *Desulfobulbacaea* depends on the surrounding geochemical environments created by other microorganisms or engineered systems. For instance, within the methanotrophic consortia, the sulfate-reducing *Desulfobulbacaea* rely on the DEET from their methane-oxidizing archaeal partners [4,36,37]. Culturing these *Desulfobulbacaea* in a purified manner may require the use of artificial electron shuttles to decouple their energy metabolism from the syntrophic partners [38]. More recent culturing efforts have suggested that rather than enriching the target microbes in tailored synthetic media placed under environmental conditions suspected to be essential, simulating the whole environment where the target microbes have been found can be a more successful strategy in enriching and finally isolating these microbes [35]. The anode of a benthic or sediment MFC was found early on to enrich members in the family of *Desulfobulbaceae* [13]. Additionally, *Desulfobulbaceae* filaments resembling CB have been observed by microscopy on the carbon fibers serving as anodes in a benthic MFC and in a bioelectrochemical reactor incubating intertidal sediment [39,40]. The environment around the anode of a MFC can exhibit a sustained redox gradient leading to an inexhaustible surface for the energy metabolism of microorganisms that utilize DEET [41], including the *Desulfobulbaceae*.

Therefore, in the present study, we worked to understand more about the *Desulfobulbaceae* enrichments that develop in bioreactors that mimic MFCs with anodes suspended either above or within anoxic sediments. By analyzing and comparing 16S rDNA of the microbial communities from two experiments, we have found that a taxonomically distinctive group of *Desulfobulbaceae* became enriched on the anodes of both designs of bioelectrochemical reactors. Microscopy assessments of the carbon fibers that served as anodes and microbial filaments isolated from sediment within the reactors suggested that this group of *Desulfobulbaceae* likely formed filaments that share morphological features with known CB or that they co-occur with CB. This group was also present in sediment inside the bioelectrochemical reactors before the reactors were capped when strong e-SOx geochemical signatures were detected. The present study delivers the latest information about an expanding number of *Desulfobulbaceae* that can respire on the oxidative electrodes and may exhibit filamentous morphologies, revealing key conditions for their metabolic activity that may lead to further enrichment and isolation.

## 2. Materials and Methods

### 2.1. Bioelectrochemical Reactor Configuration and Operation

**Figure 1:**
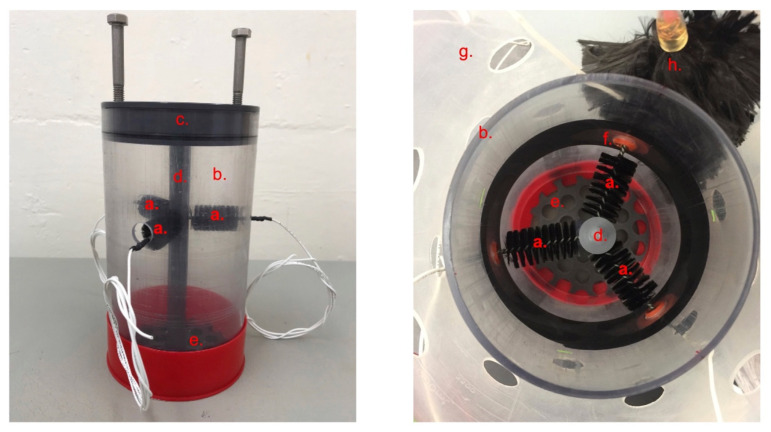
Photos of the bioelectrochemical reactor parts: (**a**) carbon brush electrodes serving as anodes, (**b**) polycarbonate core tube, (**c**) lid, (**d**) center rod, (**e**) perforated bottom partition, (**f**) septum, (**g**) perforated plastic beaker, and (**h**) cathode.

The bioelectrochemical reactors used in this study were assembled by using 15 cm long polycarbonate core tubes with 11.5 cm inner diameter (Figure 1). Sediment incorporated in the reactors was collected from Sally’s Bend, Yaquina Bay, Oregon, USA (Latitude 44°37′30″ N, Longitude 124°00′26″ W), which is about 3 km upriver from the subtidal site where the benthic MFC was positioned in the previous study [40]. The top 20 cm of cores of the muddy sediment were sieved through a 0.5 mm mesh screen and stored in a walk-in refrigerator at 5 °C. Before packing into reactors, stored sediment was first homogenized under a steady stream of nitrogen gas.

Reactor A (RA), in which the 3 electrodes were supported above the sediment–water interface, was built by packing 8 cm of sediment inside the reactor. This is the same experiment described in Li et al. (2020) [39]. Reactor B (RB), in which the 3 electrodes were set 2 cm below the sediment–water interface, was built by packing 11 cm of sediment inside the reactor. Each reactor contained three carbon brush electrodes (Mill–Rose, Mentor, OH, 8.9 cm length and 2 cm diameter) that can function as anodes. The three electrodes were inserted through septa installed in the core lining to meet predrilled holes that were positioned radially at 120° angles from each other on a polyvinyl chloride (PVC, McMaster–Carr, Elmhurst, IL, USA) center rod. A PVC lid and a perforated bottom partition were supported by the center rod as well (Figure 1).

To simulate a natural benthic environment, each reactor was set inside an 8L perforated plastic beaker that held 3 cm of intertidal sediment at the bottom. The two beakers, with the reactors inside, were gently submerged into a cooler filled with seawater collected from Yaquina Bay (salinity = 29‰). To complete the fuel cell circuit for each reactor, a carbon fiber brush electrode was placed into the seawater outside of the reactor to serve as a cathode. An Ag/AgCl electrode ([3M KCl], MI-401F, Microelectrodes, Inc., Bedford, NH, USA) was also placed in the seawater and used as a reference for monitoring the electrical potentials of anodes and the cathode within each reactor.

The two reactors were first monitored at open circuit without capping for 31 days. On day 31, the two reactors were capped to create fully anoxic conditions inside the core tubes holding sediment and seawater. It took approximately 14 and 1 day(s), respectively, for the environments around anodes of RA and RB to reach anoxic conditions. Once anoxic conditions were established, the cathode versus anode potentials were set at 300 mV using two custom-designed potentiostats (NW Metasystems, Bainbridge Island, WA, USA). This potential regulation raised anodic potentials to about 30 mV vs. the Ag/AgCl reference. In RA, one of the three electrodes inside the reactor was disconnected from the circuit to serve as a control and the other two electrodes were poised. In RB, all three electrodes inside were poised. A multichannel data logger (Agilent Technologies, Santa Clara, CA, USA, model 34970A fitted with two 34901A multiplexer modules) was used to record electrical potentials of the whole cell and the anodes (versus reference), and the current between anodes and cathode every 7 min. Seawater in the cooler was maintained at 15 °C by using a commercially available aquarium chilling system and bubbled to maintain air-saturation throughout the experiment. The cooler was covered with a black lid to prevent evaporation during operation.

Sediment samples (1 cm in length) were collected from within the reactors using cut-off syringes (0.5 cm × 3 cm) before incubation (on day 0) and before the reactors were capped (on day 31). One anode from RA was extracted through a side opening in the bioreactor tube on day 135, and one from RB on day 111. The potentials of remaining anodes in RA and RB were adjusted to ~150 mV (vs. Ag/AgCl) on day 148 and day 132, respectively. On day 184, the experiment was terminated, the two reactors were disassembled, and the remaining electrodes were sampled by trimming off bunches of carbon fibers using a pair of sterile scissors.

Sub-core samples were also taken from the sediment packed inside and outside the reactor tube (in the bottom perforated beaker). Electrode samples were frozen at −50 °C in preparation for DNA extraction and stored in fixative solution for SEM and CARD-FISH (described below). Sediment sub-core samples were frozen at −50 °C for storage and later subjected to DNA extraction. Filamentous biomass was extracted from sediment sub-cores as described in Li et al. (2020) [39].

### 2.2. Microbial Community Characterizations and Analyses

After the termination of the bioelectrochemical reactor experiments, microbial 16S rRNA community analysis was performed on 21 samples (Table S1, 2 RA anodes, 6 RB anodes, 1 RA anode before poising, 1 control electrode, 1 seeding sediment, 2 sediment inside RA, 6 sediment inside RB, 1 sediment outside RA, 1 sediment outside RB). DNeasy PowerSoil DNA Extraction Kits (Qiagen, Hilden, Germany) were used to extract the genomic DNA from all samples. Extracted DNA samples were handed off to the Center of Genome Research and Biocomputing at Oregon State University for amplification and high-throughput sequencing (Ilumina MiSeq). Primers 357wF (5′-CCTACGGGNGGCWGCAG-3′) and 785R (5′-GACTACHVGGGTATCTAATCC-3′) were chosen to amplify the V3/V4 regions of bacterial 16S rRNA genes. We used the DADA2 pipeline [42] to process our sequences either in an R package (DADA2) or Quantitative Insights into Microbial Ecology Version 2 (QIIME2) [43]. Taxonomy of each amplicon sequence variant (ASV) was classified against the Silva SSU Ref NR database (v.132). Sequences from this study are deposited in the Genbank Sequence Read Archive, under bioproject number PRJNA659795.

To assess commonality, we analyzed the common members of anodic communities in RA and RB. All obtained sequences from the anode samples of RA and RB were directly compared in Venny [44,45,46]. The ten most abundant *Desulfobulbaceae* taxa in each sample were placed in a taxonomic framework of CB [15,17] and in relation to certain family members in *Desulfobulbaceae* that are not CB. RaxML was used to construct a maximum-likelihood phylogenetic tree with 1000 bootstraps [45]. ASVs belonging to the group of uncultured *Desulfobulbaceae* were then extracted and realigned against the CB taxonomic framework to further investigate their phylogeny. To perform similarity analyses, the partial 16S rRNA gene of these ASVs was first aligned using an online multiple sequence alignment tool named MAFFT [46], and then entered into another online tool named SIAS (sequence identity and similarity, http://imed.med.ucm.es/Tools/sias.html, accessed on 23 August 2020).

### 2.3. Microscopic Examinations

Two types of samples were subjected to microscopic examination: (1) fibers of the carbon brush electrodes from inside of the reactors and (2) bacterial filaments extracted from the top one centimeter of sediment inside RA and RB after the reactors were dissembled at the end of the experiment. Fibers on the unconnected RA electrode harvested prior to the capping of reactors were used as controls.

For scanning electron microscopy (SEM) visualization, samples were first treated with a fixative that contains 2.5% glutaraldehyde and then dehydrated in a series of ethanol solutions (10 to 100%). Dehydrated specimens were mounted on aluminum SEM stubs for subsequent drying in a critical point dryer (EMS 850) and coating with gold and palladium within a sputter coater (Cressington 108). Visualization and investigation by X-ray Energy Dispersive Spectrometry (EDS) were performed by using an FEI Quanta 600FEG environmental SEM at 2–15 kV.

To perform the CARD-FISH, a *Desulfobulbaceae*-specific oligonucleotide probe (DSB706; 5′-ACC CGT ATT CCT CCC GAT-3′) modified with horseradish peroxidase (HRP) was obtained from Biomer, Germany [47]. All CARD-FISH samples were first fixed using a solution containing 2.5% glutaraldehyde. Electrode associated samples were gently washed and spread onto glass slides. Extracted filaments were retained on polycarbonate membrane filters (0.2 µm pore size) and placed onto glass slides. An agarose solution (0.2%, *w:v*) was used to mount all types of sample. The treatments of mounted samples and visualization using confocal laser scanning microscopy (CLSM) were performed as described by Li et al. (2020) [39]. By these methods, Alexa Fluro 488 (ThermoFisher, Waltham, MA, United States) was deposited along with the hybridized DSB706-HRP probe, and 4′,6-diamidino-2-phenylindole (DAPI) was applied to all samples as a counterstain.

## 3. Results

### 3.1. The Current and Voltage of the Bioelectrochemical Reactors

Signatures of e-SOx activity were observed in the top layer of sediment inside both reactors before they were capped (Li et al., 2020 [39] and Figure S1). After capping the reactors, the open circuit anodic potentials fell to about −104 mV in reactor A (RA) on day 44 and to about −350 mV in reactor B (RB) (vs. Ag/AgCl) on day 31 (Figure S2). The stabilization of different open circuit anode potentials in RA and RB suggested that anode conditions were initially more reducing in RB where the anodes contacted the sediments. By day 48, the oxygen concentration in the overlying seawater of both reactors were no longer detectable (data not shown). Once the circuit of each reactor was connected with the potentiostat, the cathode and anode potentials stabilized at approximately 330 mV and 30 mV versus Ag/AgCl, respectively. The current flow between anodes and cathodes began to rise, suggested that the anode brushes were actively accepting electrons. Anodic current collection of RA steadily increased to about 30.5 ± 2.5 µA by day 86 and then rose to about 75 ± 8 µA on day 101. After the peak, anodic current collection of RA reduced to about 30 ± 5 µA (Li et al., 2020 [39] and Figure S2). On day 135, one of the duplicate electrodes was removed, and the current collection of the leftover electrode started to increase to about 55 µA (Figure S2b), suggesting the duplicate electrodes were in competition for substrate. When the anode potential was increased to 150 mV on day 148, the current collection increased to about 58 µA and eventually stabilized at about 45 µA before we dissembled the reactor. In RB, the current collection of the triplicate electrodes was lower than the current collection from RA anodes. Current collection across each of the triplicate electrodes steadily increased to about 40.3 ± 3 µA by day 57 and then started to slowly decrease to about 33.3 ± 4 µA (Figure S2c–e). After one of the triplicate electrodes was removed on day 111, the current collections of the leftover two remained at a similar level, suggesting that the diffusion of substrate through the sediment was limiting current production (Figure S2d,e). When the anode potential was raised to 150 mV on day 132, the current collection on the remaining electrodes increased slightly and then started to decrease to about 35.5 ± 7 µA before we dissembled the reactor.

### 3.2. Microbial Communities in Bioelectrochemical Reactors

The total reads in the samples associated with the bioelectrochemical reactors (RA and RB) was 702,659. Reads varied markedly in different sample types (from 4519 to 69,734). Taxa numbering 11,146 were detected. Within anode samples, alpha diversity was higher in RB than in RA (Figure S3). A principal coordinate analysis of beta diversity indicates that RB anodes clustered closely with all sediment samples from the bioelectrochemical reactors (*p* > 0.05), and RA anodes were significantly dissimilar to the rest of our samples (*p* < 0.05, Figure S4).

**Figure 2:**
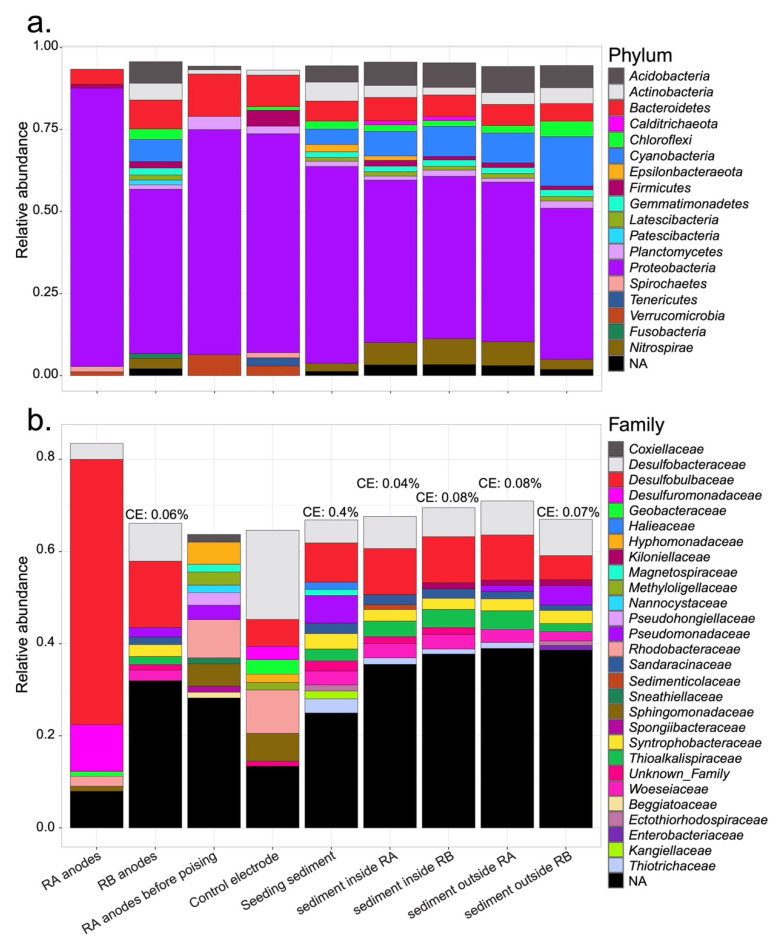
The relative abundances of (**a**) phyla and (**b**) families within *Proteobacteria* covering >1% of microbial communities. CE: a candidate cable bacteria genus, Ca. Electrothrix. NA stands for those that have not been assigned by the SILVA 132 database. Unknown Family is a family within the *Proteobacteria* that have been recognized by the SILVA 132 database but has not been given a name.

Across all samples harvested from the two bioelectrochemical reactors, *Proteobacteria* constituted the predominant phylum, ranging from 46.0 to 84.8% in relative abundance. The second most pronounced phylum was *Bacteroidetes*, ranging from 4.6 to 12.9%. *Cyanobacteria*, *Latescibacteria*, and *Nitrospirae* were seemingly more prevalent in the sediment and on the RB anodes (Figure 2a). Within *Proteobacteria,* we found that the family of *Desulfobulbaceae* was the most abundant (Figure 2b). This family composed 57.5 and 14.4% of the total relative abundance of reads from RA and RB anodes, respectively. The *Desulfuromonadaceae* family that includes members capable of respiring on an oxidative electrode was more enriched in the communities extracted from RA electrodes (10.1 and 2.9% respectively in RA anodes and control electrode). *Sandaracinaceae*, *Syntrophobacteraceae*, *Thioalkalispiraceae*, and *Woeseiaceae* were more pronounced in the sediment and on the RB anodes. The marine CB genus *Candidatus* Electrothrix was detected in certain bioelectrochemical reactor samples, ranging from 0.04% to 0.4% (Figure 2b).

**Figure 3:**
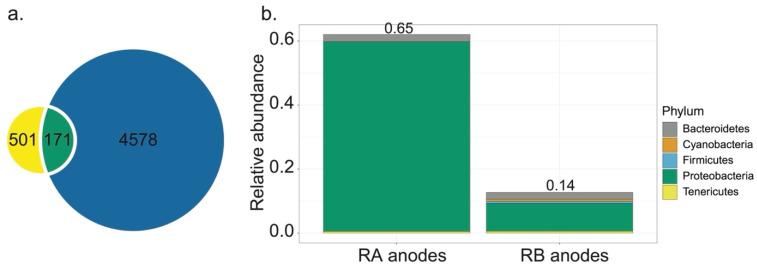
(**a**) Venn diagram of the common members of anodic communities in reactor A and B. A total of 171 amplicon sequence variants (ASVs) are shared. (**b**) The relative abundance of the shared taxa in each sample.

To investigate the potential connection between RA and RB anodes, we identified 171 ASVs that were commonly shared by the two anodic communities. These ASVs represented only 3.3% of the total number of unique ASVs detected in anode samples from the bioelectrochemical reactors (Figure 3a). However, by relative abundance, these 171 ASVs denoted 64.7 and 13.7%, respectively, of the ASVs from RA and RB anodes, suggesting that they were among the most important members of the biofilm-forming microbial community on these electrodes (Figure 3b). Within these common members, uncultured taxa within the *Desulfobulbaceae* family were highly enriched. Phylogenetic analysis indicated that a majority of the enriched uncultured *Desulfobulbaceae* taxa in our samples belong to a taxonomic group with two paraphyletic clades, C1 and C2 (Figure S5). On the partial 16S rRNA gene level, they were 93.9% similar to each other.

**Figure 4:**
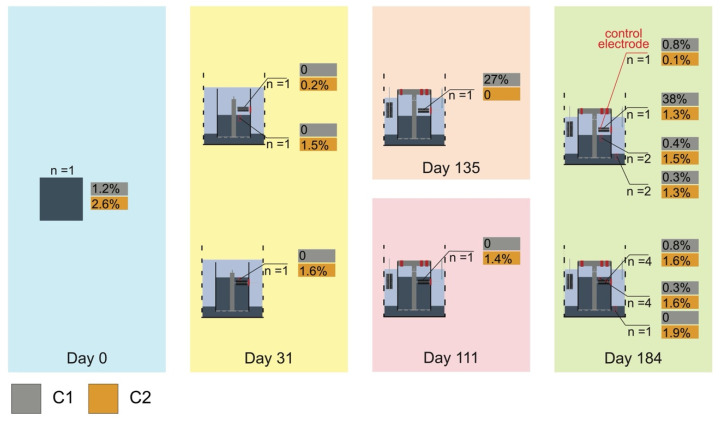
Relative abundance of the two clades of the uncultured *Desulfobulbaceae* group at different time points of bioelectrochemical reactors operation. Reactor A (RA): the 3 electrodes were hung above the sediment–water interface, displayed in the upper row. Reactor B (RB): the 3 electrodes were set below the sediment–water interface, displayed in the lower row.

We then mapped the relative abundances of these taxa over different time points of the operation of our bioelectrochemical reactors (Figure 4). We found that taxa in C1 were among the dominant members on RA anodes, 26.9 and 38.2%, respectively, at day 135 and day 184. They also appeared in the seeding sediment (1.2%), RB anodes at day 184 (0.8%), and sediment inside our reactors after cultivation (0.4 and 0.3%, respectively, in RA and RB). Taxa in C2 were enriched in the uppermost layer of sediment inside of RA and RB before the reactors were capped and at the end of our experiment, ranging from 1.5 to 1.6%. They were also in the biofilms of RA anodes at day 184 (1.3%). In the anodic biofilms of RB, C2 composed 1.6 % of the total abundance. The same group of uncultured *Desulfobulbaceae* was detected in biofilm samples from the anode of our previous benthic MFC and in the sediment associated with the burrow of *Upogebia pugettensis*, a mud shrimp species native to Yaquina Bay, Oregon [23,40].

### 3.3. Microscopic Examination of the Electrodes and Biomass inside of the Bioelectrochemical Reactors

**Figure 5:**
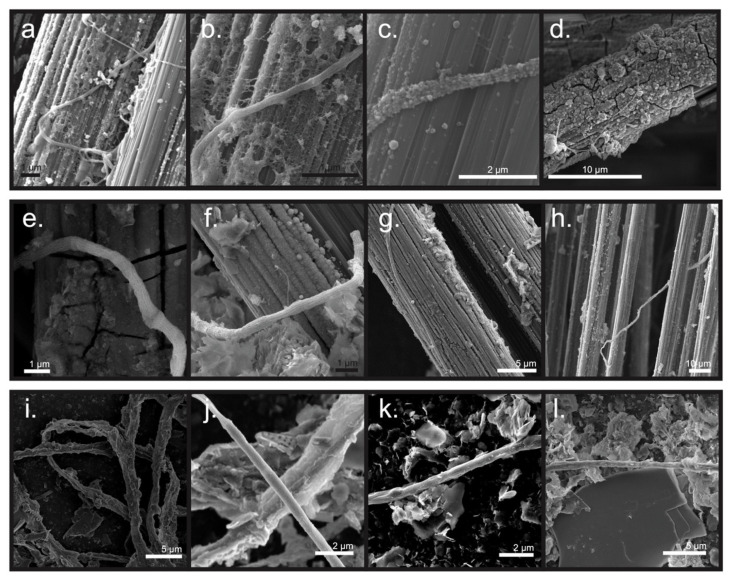
SEM images illustrating: (**a**–**d**) filamentous biomass on the surfaces of anode carbon fibers of reactor A, (**e**–**h**) filamentous biomass on and between (**h**) the surfaces of anode carbon fibers of reactor B, (**i**,**j**) filamentous biomass extracted from the uppermost sediment inside reactor A after 184 days of cultivation, and (**k**,**l**) filamentous biomass extracted from the uppermost sediment inside reactor B after 184 days of cultivation.

Microbial filaments resembling CB were observed on both carbon fibers that served as anodes of bioelectrochemical reactors and in filamentous biomass extracted from the top centimeter of sediment from inside RA and RB. For example, these filaments possessed visible cell–cell junctions (Figure 5), and longitudinal ridges were observed on several of them (Figure 5c,e,f,j). Similar to what was noted in our previous studies [23,39], the filaments possessing longitudinal ridges in the present study contained a lesser number of ridges (8 to 10) and were thinner in diameter (<1 µm) than most CB from other marine and freshwater sediment sources [16,22].

**Figure 6:**
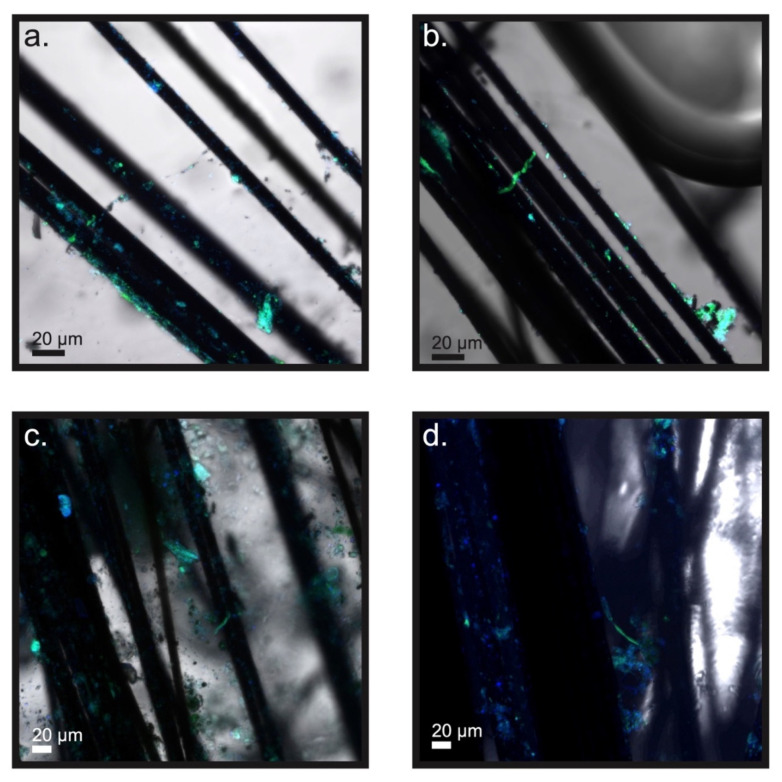
(**a**,**b**) Confocal laser scanning microscope images showing filaments and colonies belonging to the *Desulfobulbaceae* family on the surfaces of anode carbon fibers of reactor A. (**c**,**d**) Confocal laser scanning microscope images showing filaments and colonies belonging to the *Desulfobulbaceae* family on the surfaces of anode carbon fibers of reactor B. Alexa Fluor 488, green was catalytically deposited alongside with the DSB706-HRP probe and DAPI, and blue was applied as a counterstain.

Additionally, varying amounts of encrustation were found on the surface of the observed filaments. Filaments extracted from the uppermost centimeter of sediment inside the reactor tubes appeared to have the most obvious mineral encrustation (Figure 5i–l). EDS analysis showed that the coating contained oxygen (O), silicon (Si), aluminum (Al), magnesium (Mg), iron (Fe), and phosphorus (P) (12 out of 12 filaments, Figure S6). Sulfur (S) appeared on the surface of some filaments extracted from sediment inside RA (2 out of 6 filaments, Figure 6j and Figure S6c). Other studies have noted similar mineral deposits adhering to CB filaments, specifically near their oxic terminal [48]. Conversely, no mineral deposition was observed on any anode surfaces before the sealing of reactors nor on the surface of the RA control electrode that was disconnected from the circuit (though a sparse scattering of cells on the control’s carbon fibers was seen; Figure S7).

**Figure 7:**
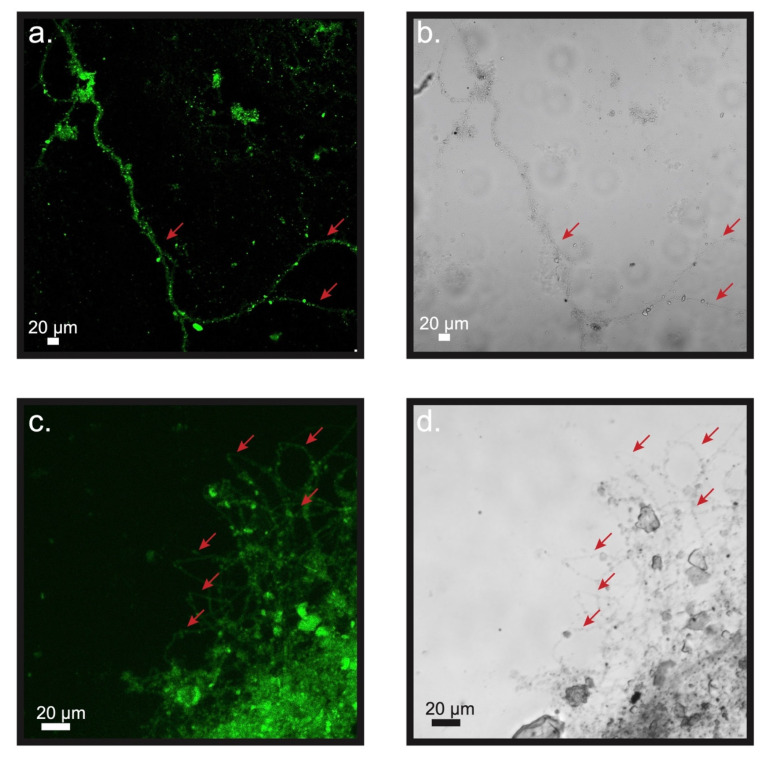
Filaments belonging to *Desulfobulbaceae* extracted from the uppermost sediment inside reactor A after 184 days of cultivation in (**a**) Alexa Fluor 488 and (**b**) bright field images; filaments belonging to *Desulfobulbaceae* extracted from the uppermost sediment inside reactor B after 184 days of cultivation in (**c**) Alexa Fluor 488 and (**d**) bright field images. Red arrows indicate the locations of filaments.

CARD-FISH revealed that the observed filaments reacted positively with the DSB706 probe universally, affirming that all forms belong to the family of *Desulfobulbaceae* (Figure 6 and Figure 7). The anodic carbon fibers also hosted various non-filamentous colonies or aggregates of cells in this family (Figure 6 and Figure 7).

## 4. Discussion

### 4.1. Enriching Novel Desulfobulbaceae by Using the Bioelectrochemical Reactors

Increasingly, members in the family of *Desulfobulbaceae* are recognized as essential agents within the biogeochemical cycles of various anoxic natural and engineered environments [9,10,11,13]. However, the roles of these microorganisms have not been thoroughly investigated, as most of this family have not been grown in isolated cultures. By using a bioelectrochemical reactor design that is like the anodic chamber of a benthic MFC, the present study has shown that certain *Desulfobulbaceae* can be enriched from sediment to become dominant on oxidative electrodes (Figure 2). Their high abundance in anode samples and the current harvested by the bioelectrochemical reactors both indicate that these *Desulfobulbaceae* will use DEET to facilitate their energy metabolism to poised electrodes (Figure 4).

The dominant presence of the C1 taxa of the *Desulfobulbaceae* on the RA anodes also suggests that the above-sediment anode configuration of the RA reactor may favor the selection of microorganisms that can disperse through water and survive on the surface of an oxidative electrode (Figure 4). Several members in the *Desulfobulbaceae* family including CB can perform chemotaxis [6,49,50]. Other causes of differences in observed diversity indexes and microbial community compositions between RA and RB were likely the geochemical environment around the anodes and access to different forms of organic matter. The environment around the RA anodes is surmised to have shaped the community to rely on the oxidation of dissolved organic matter diffusing out of the sediment and reduction of the oxidative electrodes. Additionally, soluble electron shuttles released from the sediment such as iron and sulfur species were likely also oxidized and re-reduced on the oxidative electrode surface [51]. Therefore, a less diverse microbial community, dominated by bacteria that are electrogenically active and participate in iron/sulfur cycling, was found. In contrast, RB anodes were positioned within an intertidal sediment matrix replete with natural particulate organic matter, minerals, and electron shuttles such as elemental sulfur and humic substances. Diverse microorganisms in intertidal sediment can break down organic matter under anoxic conditions using a range of electron shuttles as electron acceptors [52]. Reduced electron shuttles can further transfer electrons to conductive minerals like magnetite and ferric oxide biotically or abiotically [53,54]. The presence of oxidative electrodes functioned as the final electron acceptors whose surface was continuously accepting electrons from electrogenic bacteria, reduced electron shuttles, and reduced minerals, enhancing the overall reaction rate. Therefore, a more diverse microbial community with members able to degrade complex organic matter and reduce minerals occurring in the sediment is indicated, as well as members able to assimilate and metabolize low molecular weight dissolved organic matter products.

### 4.2. Desulfobulbaceae Observed inside the Bioelectrochemical Reactors

The microscopic results in the present study indicate that filaments of *Desulfobacteraceae* were present in the bioelectrochemical reactors. Though Ca. Electrothrix, a known genus of CB, was detected in the microbial 16S rRNA communities of RA and RB samples before and at the end of the experiment, and the relative numbers of reads of Ca. Electrothrix were low, even in sediments that exhibited strong geochemical signatures of e-SOx (Figure 2 and Figure S2). In lab-cultured subtidal sediments collected from Aarhus Bay, Denmark, Ca. Electrothrix have comprised up to 8% of the total bacteria and up to 64% of the *Desulfobulbaceae* population [49]. In our samples, the relative abundance of Ca. Electrothrix only represented 0 to 4.7% of the *Desulfobulbaceae* population (Figure 2). A study of the microbial community composition using the same sequencing technique as the present study found Ca. Electrothrix abundances of only 0.01 to 0.3% in sediments associated with the burrows of *U. pugettensis* collected from another intertidal mudflat in Yaquina Bay after 40 days of culture [23]. Therefore, it is likely that low abundances of Ca. Electrothrix may be typical in intertidal mudflat sediments collected from Yaquina Bay and other similar Northwest American estuaries. Additionally, a recent study indicated the possible existence of another genus of CB in sediments under brackish waters with salinities above 7 and below 35‰ [55]. It should be noted that the intertidal mudflat where we collected our sediment was approximately 5 km away from the mouth of the Yaquina Bay estuary and subjected to seasonal influences from the freshwater discharge of the Yaquina River [56]. The salinity is typically 13 to 32‰ in the wet season and 28 to 34‰ in the dry season throughout the lower estuary. Therefore, we suspect that the observed Ca. Electrothrix abundance alone did not account for the e-SOx activity and filaments observed in this study.

Several observations lead to a hypothesis that the filamentous *Desulfobulbaceae* biomass observed in the bioelectrochemical reactors are rightfully described as “CB”, but they belong to a unique taxonomic group containing C1 and C2. Firstly, many of these filaments possessed the unique morphological features of iconic CB. Unique morphological features, namely, filamentous form, cell–cell junctions, and longitudinal ridges, are considered as required characteristics of CB that enable DEET via e-SOx [33,34]. Therefore, the identification of CB has often relied on microscopic observations of these distinctive features, combined with fluorescence in situ hybridization labeling and observation of the geochemical signatures of e-SOx [14,24,25]. Strong geochemical signatures did exist in the uppermost sediment of both RA and RB reactors before the reactors were capped (Figure S2). Secondly, it seems that these *Desulfobulbaceae* especially those identified as C1 will disperse into an anaerobic water column to look for suitable electron acceptors as will previously recognized CB. Abilities to encode periplasmic cytochromes and survive on oxidative electrodes imply that CB are electrogenic bacteria [39,57]. Taxa in C1 and C2 coexist naturally in the intertidal sediment from Yaquina Bay, Oregon, and they may proliferate according to their metabolic preferences and conditions when cultured in bioelectrochemical reactors.

The findings of the present study shed new light on the results of our previous studies that concluded that CB can be attracted to and survive on electrodes poised at an oxidative potential [39]. Though more work is still needed to further study these new taxa in enrichment cultures and to establish their relationship to other CB and family members of *Desulfobulbaceae*, our results suggest that many *Desulfobulbaceae* may form cellular cables. Despite having no gene to encode oxygen reductase, oxygen is still known as the most favorable electron acceptor for marine and freshwater CB genera, Ca. Electrothrix and Ca. Electronema [57]. Nitrate has been reported to serve as an alternative electron acceptor for CB in these two genera when oxygen is absent in the overlying seawater [58]. On the other hand, CB found within groundwater sediments seem to have broader abilities to couple a series of alternative metabolic pathways to acquire energy. For example, other than oxidizing sulfide, groundwater CB can grow via the disproportionation of elemental sulfur and may use sulfur as an electron donor and nitrate or ferrihydrites as electron acceptors [17]. SEM and EDS analyses in the present study suggest that iron or sulfur cycling may also be important to the metabolic strategy of the CB filaments we have found on anodes or inside anaerobic reactor sediments (Figure S6). Interestingly, an early benthic MFC study pointed out strong geochemical evidence for the existence of sulfur-iron cycling associated with anode biofilms [51]. The redox-gradient microenvironment created by the presence of an oxidative electrode is concluded to be favorable to CB under otherwise anoxic conditions. It should also be noted that in the present study, the CB filaments observed on the electrode surface were generally short (<1000 µm) and thin (<1 µm). The redox environment appears to play an important role in controlling the length and morphology of CB. For example, in sediments heavily bioturbated by the tubeworm *Chaetopterus variopedatus* and mud shrimp *U. pugettensis*, where redox conditions often oscillate between oxic and hypoxic, CB were present predominately in short filaments [20,23]. Thinner filaments and individual cells with no longitudinal ridges also have been observed when growing groundwater CB in anaerobic bottles with sulfur and nitrate [17]. In concert, these observations indicate that changes in the redox environment may reduce the advantage of forming long filaments or longitudinal ridges.

## 5. Conclusions

Many DEET-utilizing microorganisms contribute to the Earth’s microbial diversity, but few have been directly cultured using traditional microbiological tools and strategies [59,60]. The “unculturable microorganisms” may require alternative culture methods that mimic environmental factors [35]. The present study has demonstrated the possibility of using bioelectrochemical reactors to enrich a group of novel *Desulfobulbaceae* that were observed to form filaments and respire on the oxidative electrodes. Further enriching these *Desulfobulbaceae* will not only help us to learn their role in ecology and elemental cycling but also may provide a successful strategy to fully isolate these and other electrogenic taxa that can perform DEET.

This study was also conducted, in part, to understand how CB would interact with an oxidative electrode surface. Though more effort is still needed to develop means to identify filaments observed in the bioelectrochemical reactors as CB with complete certainty, we can affirm that CB survived under anoxic conditions with the presence an oxidative electrode, and they with other *Desulfobulbaceae* likely shuttled electrons directly or indirectly to the oxidative electrode. This observation provides critical information on how populations of CB may survive in various habitats with low oxygen concentration such as those that are within the ocean’s oxygen minimum zone and inside of the anodic chamber of a benthic MFC. Taken together with the recent findings of new CB in groundwater aquifer sediment and sediment with pore waters at intermediate salinity, it is likely that there are probably other (perhaps many) filamentous members of *Desulfobulbaceae* that can perform e-SOx and may be called CB. Further identification of these members not only contributes to the growing body of knowledge of CB phylogeny, but it may also lead to all members being enriched and isolated successfully.

## Data Availability

Sequences from this study are deposited in the Genbank Sequence Read Archive, under bioproject number PRJNA659795.

## Supplementary Materials

The following are available online at https://www.mdpi.com/article/10.3390/microorganisms9112329/s1, Figure S1: Representative microelectrode depth profiles of oxygen (blue), pH (red), and ΣH_2_S (orange) in bioelectrochemical reactor B at day 13 and day 24, Figure S2. The current production (blue), the anodic potential (black), and cathodic potential (orange) over time during the bioelectrochemical reactor experiments. Because there were three anode electrodes and a single cathode in each reactor, the cathode potential is the same across records linked to shared reactors. In (a) & (b) the results are from duplicate poised electrodes in bioelectrochemical reactor A and are the same measurements as reported in Li et al. 2020. In (c), (d), & (e) the records are from bioelectrochemical reactor B which had triplicate poised anodes. The reference electrode was an Ag/AgCl electrode with 3M KCl filling solution, Figure S3. Alpha diversity measures of observed ASVs, Chao1, and Shannon indexes for anode samples harvested at different times from the bioelectrochemical reactors and compared to multiple samples from the BMFC sampled in 2015. Observed ASVs and Chao1 measure the true species diversity whereas the Shannon index incorporates both richness and evenness. Conditions: RA, reactor A; RB, reactor B; BMFC, harvested from previous BMFC in 2015; 150 mV and 30 mV, the poised potentials prior to harvest, Figure S4. Principal coordinate analysis (PCoA) based on unweighted Unifrac distance for all samples. Conditions: BMFC2015, harvested from previous BMFC in 2015, Figure S6. X-Ray Energy Dispersive Spectrometry (EDS) spectra from areas of cable bacteria filaments. The inserts show scanning electron microscopy (SEM) images of the targeted filaments. Samples were coated with gold and palladium before visualization. Sample (a) is a filament on an anode carbon fiber from bioelectrochemical reactor A; (b) is from an anode sample of bioelectrochemical reactor B; (c) shows a filament separated from the sediment in bioelectrochemical reactor A; and (d) is a filament with surrounding sediment from bioelectrochemical reactor B, Figure S7. Scanning electron microscopy (SEM) images of carbon fibers of control electrodes (a) harvested before reactors were capped on day 31, and (b) after the reactors were dissembled on day 184, Table S1: Information of samples in reactor A (RA) and reactor B (RB).
