# Supplementary material for: Using Oxidative Electrodes to Enrich Novel Members in the Desulfobulbaceae Family from Intertidal Sediments
Source: Microorganisms. 2021 Nov 11;9(11):2329. doi: 10.3390/microorganisms9112329 (PMC8618199; doi:10.3390/microorganisms9112329)
Supplement: Supplementary file 1 [file microorganisms-09-02329-s001.zip › microorganisms-1370223-supplementary.pdf]

*Supplementary Materials*

# Using Oxidative Electrodes to Enrich Novel Members in the *Desulfobulbaceae* Family from Intertidal Sediments

**Cheng Li *, Clare E. Reimers and Yvan Alleau**

College of Earth, Ocean, and Atmospheric Sciences, Oregon State University, Corvallis, OR 97331, USA; Clare.Reimers@oregonstate.edu (C.E.R.); yvan.alleau@oregonstate.edu (Y.A.)
* Correspondence: cheng.li@oregonstate.edu

## 1. Microelectrode Measurements of e-SOx Geochemical Signatures

Geochemical signatures of e-SOx, the two pH maximums and the separation of oxygen penetration and sulfide reappearing depths, were measured on days 13 and 24 of reactor incubation using commercial microelectrodes (tip size = 100 μm, Unisense A.S., Aarhus, Denmark) operated with a field multimeter and positioned with a micromanipulator. For both reactor A (RA) and reactor B (RB), profiling was done by lowering the microelectrodes through the carbon fiber brushes while avoiding the middle titanium-wire core. On day 48, circuits in RA and RB were disconnected and microelectrode profiling was repeated by lowering the sensors through the ports in the reactor lid. The pH microelectrode was broken when profiling RA and therefore no pH or calculated total sulfide are reported. The calibration and profiling of pH, $H_2S$, and $O_2$ microelectrode were performed as described by [1].

During initial open-circuit incubations of RA and RB, the top centimeter of sediment in each reactor changed from dark to light gray. Oxygen penetration depths and sulfide reappearing depths were observed to separate when profiling with microelectrodes after 13 days of culture (Fig. S2). Total sulfide concentrations were low compared to previous studies of marine sediments hosting cable bacteria [2]. No subsurface pH maximum was detected, but a pH minimum of about 7.5 occurred at ~8 mm depth in both reactors.

Signatures of e-SOx activity were more pronounced after 24 days of culture. Not only did the oxygen penetration and sulfide reappearing depths separate further, but there were also distinctive subsurface pH maxima (8.7 to 8.8 in RA and 8.7 to 9.0 in RB) and pH minimums (6.8 to 7.0 in RA and 6.6 to 6.8 in RB) in the deeper sediment (9.6 to 10.4 mm in RA and 10.4 to 14 mm in RB). These observations suggest that e-SOx activity had been established in the uppermost layer of sediment within the reactors before the reactors were sealed to create anoxic conditions, 31 days into the incubations.

When microelectrode profiling was performed a third time on day 48, anodes in both RA and RB were actively serving as electron acceptors, and the microelectrode measurements confirmed that the overlying seawater was anoxic except right below the sample port. At this time point, the sulfide reappearing depth in sediment inside of RA and RB had decreased from 10.7 to 0 mm and 14 to 8.9 mm, respectively. The subsurface pH maximum was also no longer detected in sediment inside RB (while no pH measurements

were made in RA). These observations indicate that the vertical geochemical signature of e-SOx in the sediments had been altered.

**2. Calculation of alpha and beta diversity**

Through the built-in core metric phylogenetic method in Quantitative Insights into Microbial Ecology Version 2 (QIIME2) [3], we analyzed the alpha and beta diversity by rarefying our samples on a sequence depth of 4,500, the lowest number among all of our samples. Alpha and beta diversity was then illustrated respectively using an R package phyloseq (v.1.30.0) [4] and using the EMPeror [5].

**Table S1**. Information of samples in reactor A (RA) and reactor B (RB).

| Sample number | Position | Type | The poised potentials prior to harvest* | Time from start of incubation | Sample date (yyyy-mm-dd) |
|---|---|---|---|---|---|
| S050 | RA anodes | Anode | 30 mV | Day 135 | 2018-10-22 |
| S051 | RB anodes | Anode | 30 mV | Day 111 | 2018-09-28 |
| S052 | RB anodes | Anode | 30 mV | Day 111 | 2018-09-28 |
| S053 | RA anodes before poising | Control | N/A | Day 31 | 2018-07-10 |
| S054 | Sediment inside RA | Sediment | N/A | Day 31 | 2018-07-10 |
| S055 | Sediment inside RB | Sediment | N/A | Day 31 | 2018-07-10 |
| S056 | Seeding sediment | Sediment | N/A | Day 0 | 2018-06-09 |
| S057 | RA anodes | Anode | 150 mV | Day 184 | 2018-12-20 |
| S058 | Control electrode | Control | N/A | Day 184 | 2018-12-20 |
| S059 | Sediment inside RA | Sediment | N/A | Day 184 | 2018-12-20 |
| S060 | Sediment inside RB | Sediment | N/A | Day 184 | 2018-12-20 |
| S061 | Sediment outside RA | Sediment | N/A | Day 184 | 2018-12-20 |
| S062 | RB anodes | Anode | 150 mV | Day 184 | 2018-12-20 |
| S063 | RB anodes | Anode | 150 mV | Day 184 | 2018-12-20 |
| S064 | Sediment inside RB | Sediment | N/A | Day 184 | 2018-12-20 |
| S065 | Sediment inside RB | Sediment | N/A | Day 184 | 2018-12-20 |
| S066 | Sediment outside RB | Sediment | N/A | Day 184 | 2018-12-20 |
| S067 | Sediment inside RB | Sediment | N/A | Day 184 | 2018-12-20 |
| S068 | Sediment inside RB | Sediment | N/A | Day 184 | 2018-12-20 |
| S070 | RB anodes | Anode | 150 mV | Day 184 | 2018-12-20 |
| S071 | RB anodes | Anode | 150 mV | Day 184 | 2018-12-20 |

* Potential was measured against Ag/AgCl reference electrode (in 3M KCl).

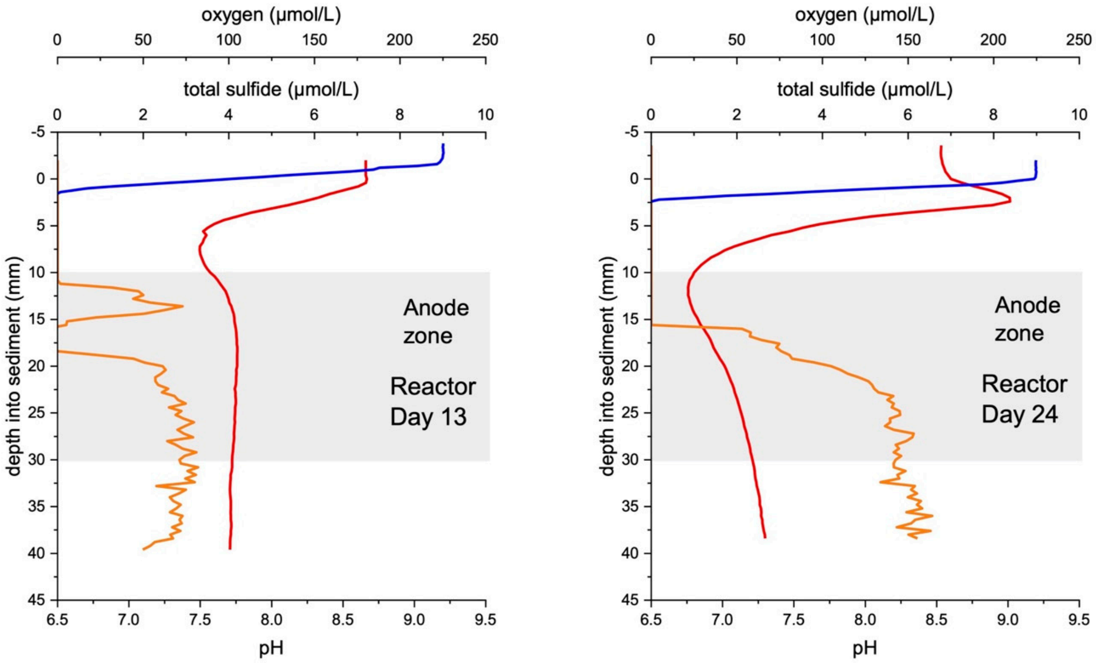


**Figure S1**. Representative microelectrode depth profiles of oxygen (blue), pH (red), and $\Sigma H_2S$ (orange) in bioelectrochemical reactor B at day 13 and day 24.

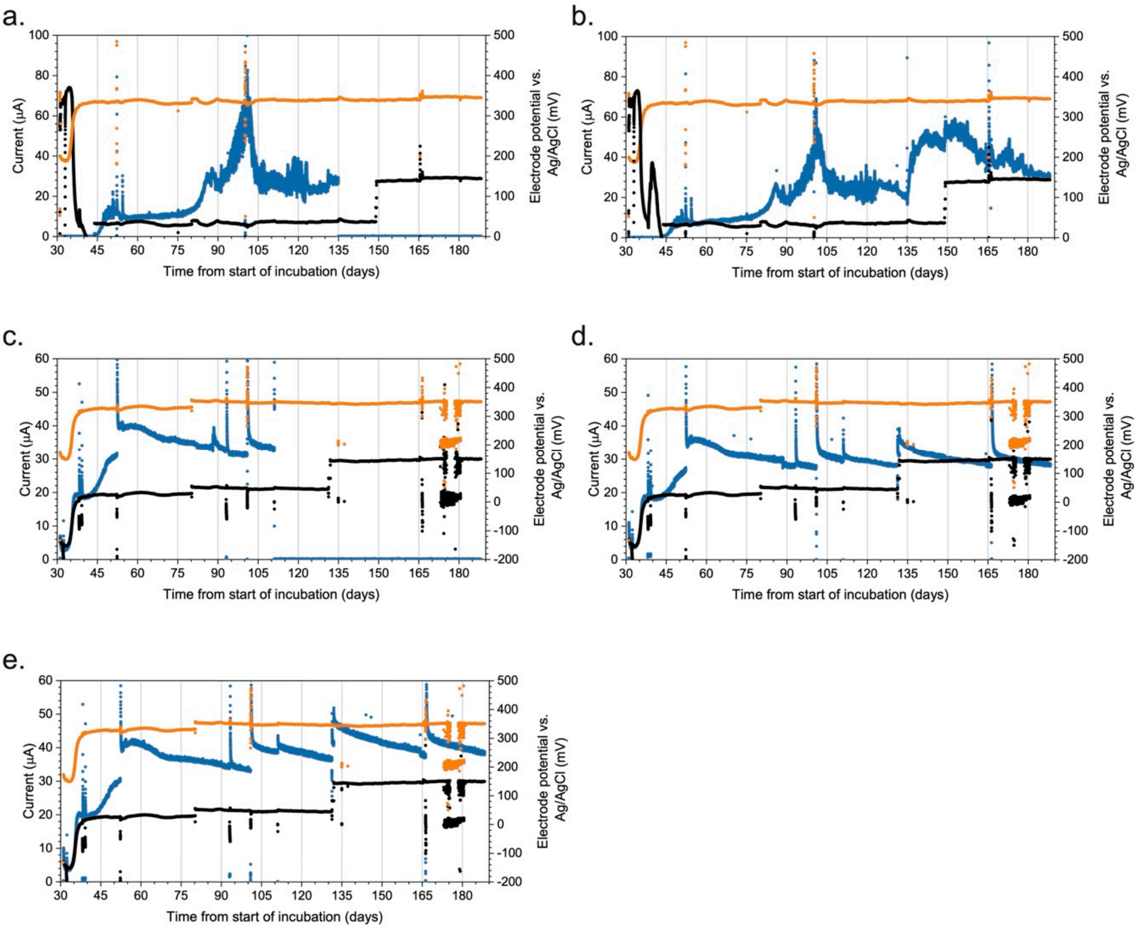


**Figure S2**. The current production (blue), the anodic potential (black), and cathodic potential (orange) over time during the bioelectrochemical reactor experiments. Because there were three anode electrodes and a single cathode in each reactor, the cathode potential is the same across records linked to shared reactors. In (a) & (b) the results are from duplicate poised electrodes in bioelectrochemical reactor A and are the same measurements as reported in Li et al. 2020. In (c), (d), & (e) the records are from bioelectrochemical reactor B which had triplicate poised anodes. The reference electrode was an Ag/AgCl electrode with 3M KCl filling solution.

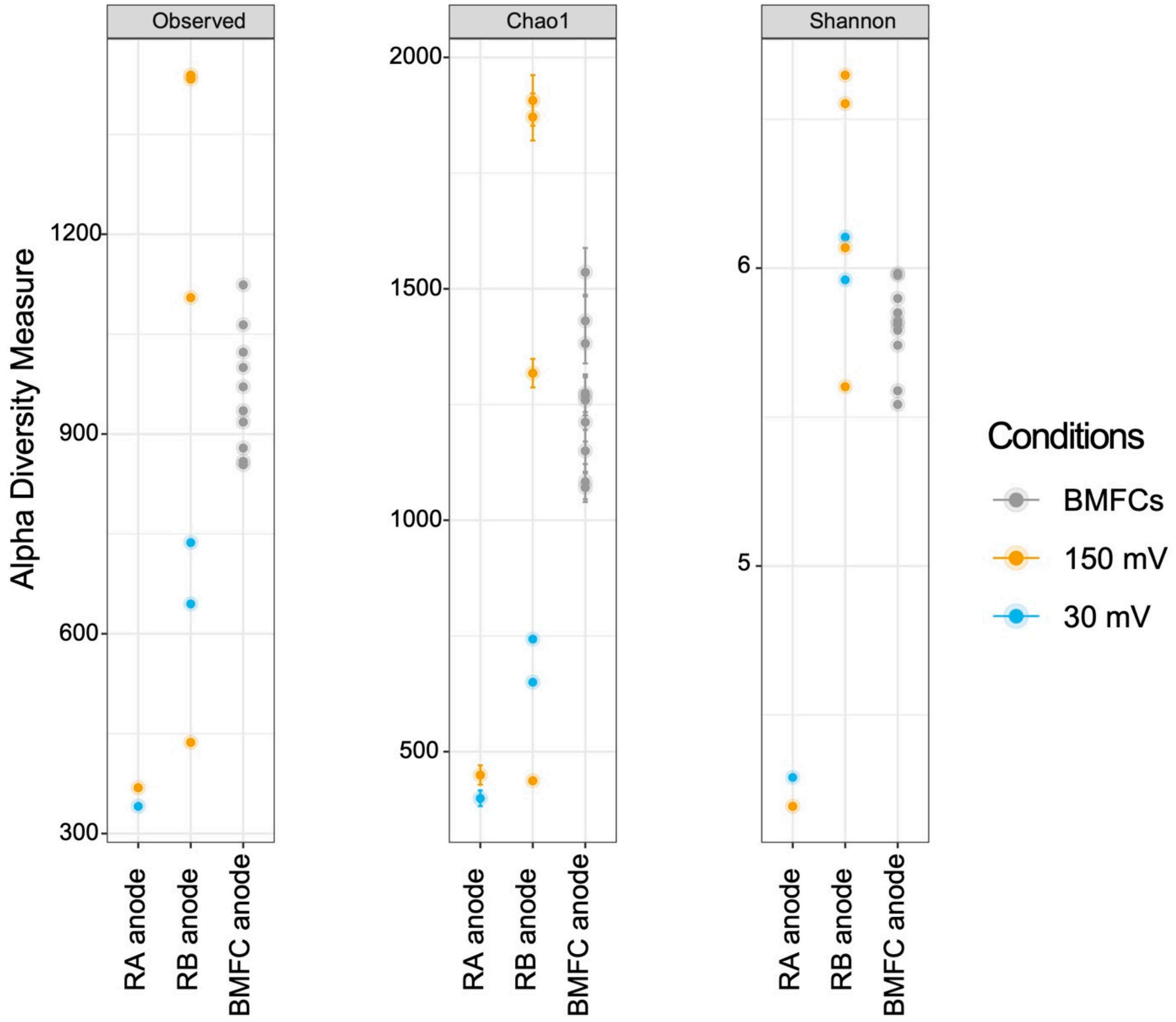


**Figure S3**. Alpha diversity measures of observed ASVs, Chao1, and Shannon indexes for anode samples harvested at different times from the bioelectrochemical reactors and compared to multiple samples from the BMFC sampled in 2015 [5]. Observed ASVs and Chao1 measure the true species diversity whereas the Shannon index incorporates both richness and evenness. Conditions: RA, reactor A; RB, reactor B; BMFC, harvested from previous BMFC in 2015; 150 mV and 30 mV, the poised potentials prior to harvest.

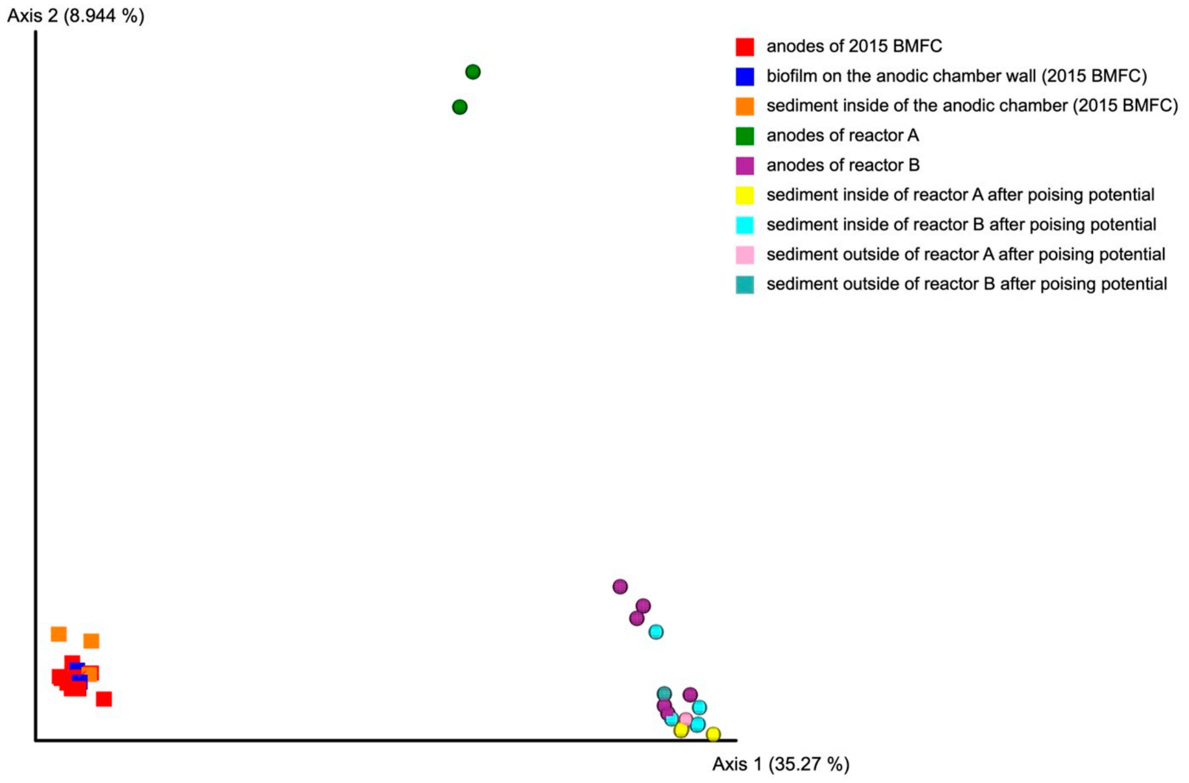


**Figure S4.** Principal coordinate analysis (PCoA) based on unweighted Unifrac distance for all samples. Conditions: BMFC2015, harvested from previous BMFC in 2015 [5].

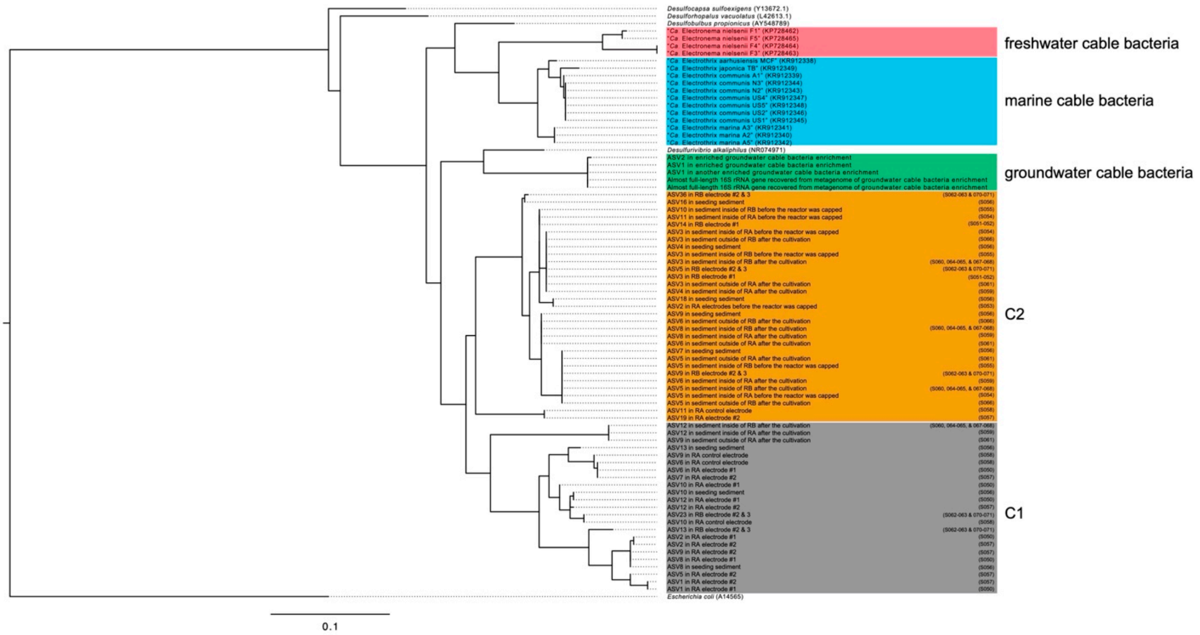


**Figure S5.** Phylogenetic tree based on the *Desulfobulbaceae* 16s rRNA gene sequences recovered from the samples within bioelectrochemical reactor A and B. Color boxes indicate previously recognized genera of cable bacteria [6,7] and the taxonomic group of uncultured *Desulfobulbaceae*. C1: clade 1 and C2: clade 2. Scale bar shows 10% sequence divergence. Numbers indicate the sample number in Table S1.

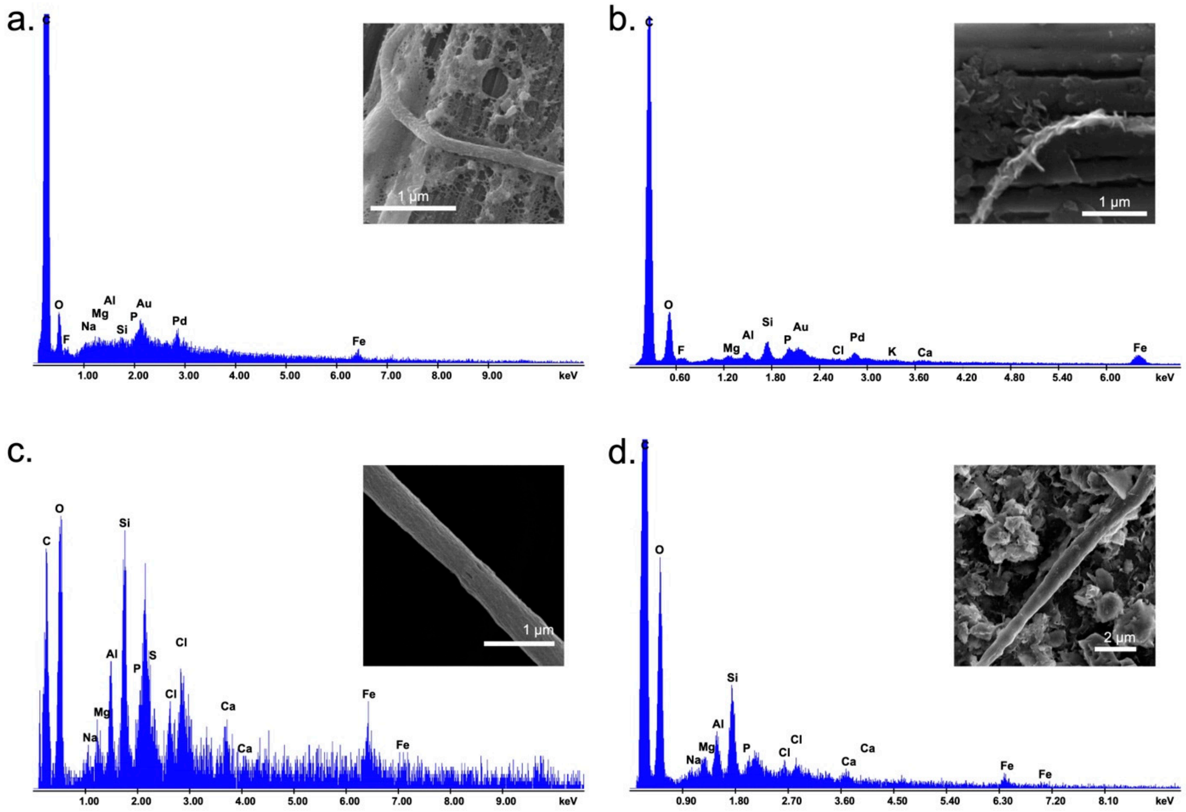


**Figure S6.** X-ray Energy Dispersive Spectrometry (EDS) spectra from areas of cable bacteria filaments. The inserts show scanning electron microscopy (SEM) images of the targeted filaments. Samples were coated with gold and palladium before visualization. Sample (a) is a filament on an anode carbon fiber from bioelectrochemical reactor A; (b) is from an anode sample of bioelectrochemical reactor B; (c) shows a filament separated from the sediment in bioelectrochemical reactor A; and (d) is a filament with surrounding sediment from bioelectrochemical reactor B.

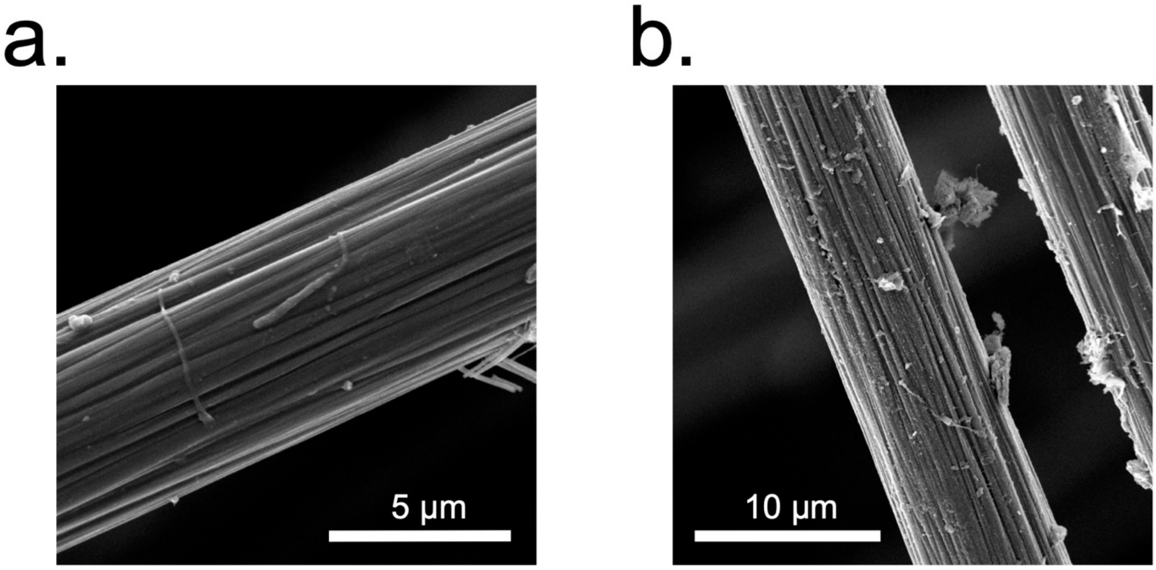


**Figure S7.** Scanning electron microscopy (SEM) images of carbon fibers of control electrodes a) harvested before reactors were capped on day 31, and b) after the reactors were dissembled on day 184.

**References**

1. Li, C.; Reimers, C.E.; Alleau, Y. Inducing the Attachment of Cable Bacteria on Oxidizing Electrodes. *Biogeosciences* 2020, *17*, 597–607, doi:10.5194/bg-17-597-2020.
2. Bolyen, E.; Rideout, J.R.; Dillon, M.R.; Bokulich, N.A.; Abnet, C.C.; Al-Ghalith, G.A.; Alexander, H.; Alm, E.J.; Arumugam, M.; Asnicar, F.; et al. Reproducible, Interactive, Scalable and Extensible Microbiome Data Science Using QIIME 2. *Nat Biotechnol* 2019, *37*, 852--857, doi:10.1038/s41587-019-0209-9.
3. McMurdie, P.J.; Holmes, S. Phyloseq: An R Package for Reproducible Interactive Analysis and Graphics of Microbiome Census Data. *PLOS ONE* 2013, *8*, e61217.
4. Vazquez-Baeza, Y.; Pirrung, M.; Gonzalez, A.; Knight, R. EMPeror: A Tool for Visualizing High-Throughput Microbial Community Data. *Gigascience* 2013, *2*, doi:10.1186/2047-217x-2-16.
5. Reimers, C.E.; Li, C.; Graw, M.F.; Schrader, P.S.; Wolf, M. The Identification of Cable Bacteria Attached to the Anode of a Benthic Microbial Fuel Cell: Evidence of Long Distance Extracellular Electron Transport to Electrodes. *Front Microbiol* 2017, *8*, doi:10.3389/fmicb.2017.02055.
6. Trojan, D.; Schreiber, L.; Bjerg, J.T.; Bggild, A.; Yang, T.; Kjeldsen, K.U.; Schramm, A. A Taxonomic Framework for Cable Bacteria and Proposal of the Candidate Genera Electrothrix and Electronema. *Syst Appl Microbiol* 2016, *39*, 297--306, doi:10.1016/j.syapm.2016.05.006.
7. Müller, H.; Marozava, S.; Probst, A.J.; Meckenstock, R.U. Groundwater Cable Bacteria Conserve Energy by Sulfur Disproportionation. *Isme J* 2020, *14*, 623–634, doi:10.1038/s41396-019-0554-1.
